# Sex Differences in Tau Biomarkers and Brain Functional Connectivity in Alzheimer’s Disease

**DOI:** 10.1002/alz.094898

**Published:** 2025-01-09

**Authors:** Jack Lei Yang, Jordan Williamson, Yuan Yang

**Affiliations:** ^1^ Carle Illinois College of Medicine, Urbana‐Champaign, IL USA; ^2^ University of Illinois Urbana‐Champaign, Urbana‐Champaign, IL USA

## Abstract

**Background:**

As of 2024, an estimated 6.9 million individuals reside in the United States with Alzheimer’s Disease (AD). Previous studies suggest that AD disproportionately affects females, who exhibit a higher incidence rate, poorer performance on various neuropsychological tasks, and more significant total brain atrophy. Recent investigations reveal differences in hippocampal functional connectivity based on sex, potentially contributing to the observed sex‐related disparities in AD. Moreover, individuals carrying higher levels of the tau protein show reduced hippocampal functional connectivity.

**Method:**

Using Resting State fMRI and T2 MRI data from participants with AD (n = 20, female = 9) and cognitively normal individuals (n = 20, female = 9) from the Alzheimer’s Disease Neuroimaging Initiative (ADNI), we employed the Functional Connectivity Toolbox (CONN) for analysis.

**Result:**

Our findings revealed differences in hippocampal functional connectivity between individuals with higher and lower levels of plasma NT1‐tau levels in each group. Additionally, sex differences in hippocampal functional connectivity were observed among AD participants with higher tau protein levels.

**Conclusion:**

These results enhance our understanding of the complex interplay between sex, tau levels, and hippocampal function in AD. Such insights into biomarkers may inform the development of sex‐specific interventions aimed at enhancing AD treatment efficacy.

